# Using ordinal logistic regression to evaluate the performance of laser-Doppler predictions of burn-healing time

**DOI:** 10.1186/1471-2288-9-11

**Published:** 2009-02-16

**Authors:** Rose D Baker, Christian Weinand, James C Jeng, Henk Hoeksema, Stan Monstrey, Sarah A Pape, Robert Spence, David Wilson

**Affiliations:** 1 Centre for Operational Research and Applied Statistics, University of Salford, Salford, M5 4WT, UK; 2 The Washington Hospital Center, Laboratory for Burn and Tissue Regeneration, Washington, DC, USA; 3 Department of Plastic Surgery, University Hospital Ghent, Ghent, Belgium; 4 Northern Regional Burns Network, Royal Victoria Infirmary, Newcastle upon Tyne, UK; 5 Burn Surgery, Johns Hopkins School of Medicine, Baltimore, Maryland, USA; 6 Plastic Surgery, Burns Unit, Nottingham CityHospital, Nottingham, UK

## Abstract

**Background:**

Laser-Doppler imaging (LDI) of cutaneous blood flow is beginning to be used by burn surgeons to predict the healing time of burn wounds; predicted healing time is used to determine wound treatment as either dressings or surgery. In this paper, we do a statistical analysis of the performance of the technique.

**Methods:**

We used data from a study carried out by five burn centers: LDI was done once between days 2 to 5 post burn, and healing was assessed at both 14 days and 21 days post burn. Random-effects ordinal logistic regression and other models such as the continuation ratio model were used to model healing-time as a function of the LDI data, and of demographic and wound history variables. Statistical methods were also used to study the false-color palette, which enables the laser-Doppler imager to be used by clinicians as a decision-support tool.

**Results:**

Overall performance is that diagnoses are over 90% correct. Related questions addressed were what was the best blood flow summary statistic and whether, given the blood flow measurements, demographic and observational variables had any additional predictive power (age, sex, race, % total body surface area burned (%TBSA), site and cause of burn, day of LDI scan, burn center). It was found that mean laser-Doppler flux over a wound area was the best statistic, and that, given the same mean flux, women recover slightly more slowly than men. Further, the likely degradation in predictive performance on moving to a patient group with larger %TBSA than those in the data sample was studied, and shown to be small.

**Conclusion:**

Modeling healing time is a complex statistical problem, with random effects due to multiple burn areas per individual, and censoring caused by patients missing hospital visits and undergoing surgery. This analysis applies state-of-the art statistical methods such as the bootstrap and permutation tests to a medical problem of topical interest. New medical findings are that age and %TBSA are not important predictors of healing time when the LDI results are known, whereas gender does influence recovery time, even when blood flow is controlled for.

The conclusion regarding the palette is that an optimum three-color palette can be chosen 'automatically', but the optimum choice of a 5-color palette cannot be made solely by optimizing the percentage of correct diagnoses.

## Background

The use of laser-Doppler imaging (LDI) for predicting burn healing time is increasing, and there are several recent reviews that compare it favorably with other techniques ([1-4]). The basis of the methodology is that the proximate cause of healing is skin blood flow, which is measured by the laser-Doppler imager in perfusion units (PU).

The medical decision to be made is whether to allow healing to occur naturally, or, if that would be too slow, to operate to excise the burn and apply a skin graft. As a decision aid, clinicians consider it desirable to predict healing time as being either less than 14 days, 14–21 days, or over 21 days. Hence model predictions are ordinal. There are many papers describing the benefits of LDI as a decision aid for clinicians, e.g. [5-8]. In essence, LDI can enable predictions of healing time that are correct over 90% of the time, whereas unaided clinician judgement is correct only about 70% of the time [5].

**Figure 1:**
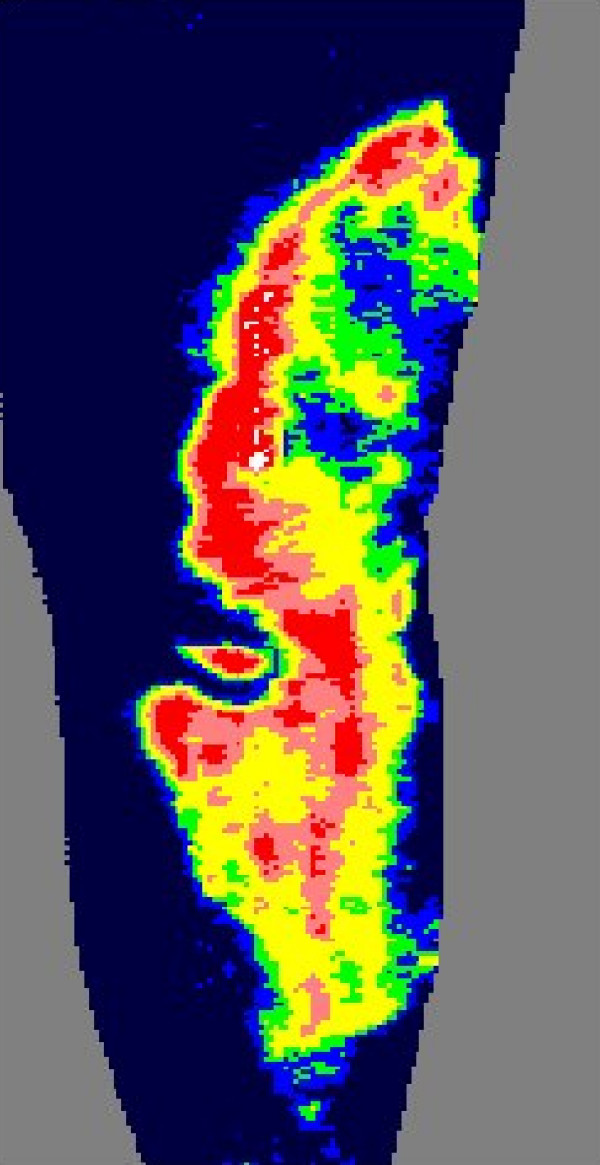
**Picture of a laser-Doppler burn image**.

**Figure 2:**
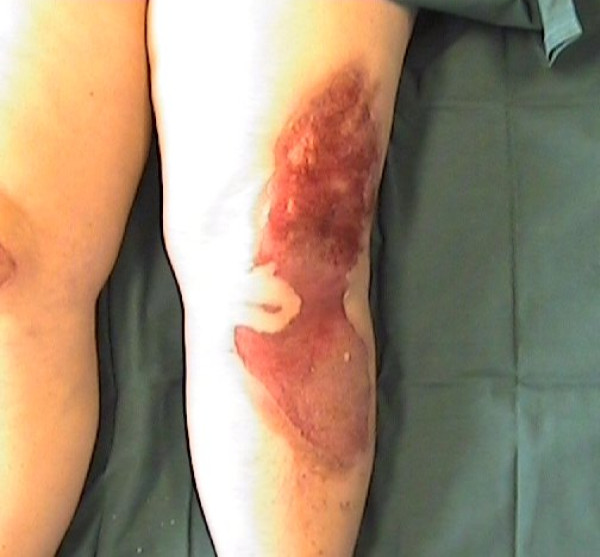
**Color photograph of the same burn area**.

This paper uses data collected by five burn centers, two in the UK, one in Belgium and two in the USA, to develop and validate a probabilistic model of burn healing time, as a function of the laser-Doppler flux measurements, and of demographic and observational variables. Besides confirming the performance of laser-Doppler methodology, such modeling can address the question of its statistical sufficiency. This is, whether, given laser-Doppler blood flow measurements, demographic and observational variables have any further predictive ability, or whether the blood flow measurement recorded as mean flux in PU is a sufficient statistic (see [9] for a definition of this term). The model also allows 'what if' questions to be asked, such as how far predictive ability might degrade on moving to a different patient mix than that seen in the data, for example for patients with a much higher percentage of total burned surface area (%TBSA). Current practice is for the clinician to view a false-color image of the region of interest, in which high blood flow areas are colored red and low blood flow regions blue. Figures 1 and 2 give an example. A suitable color palette must be chosen for this purpose, and this problem is also discussed here.

We may expect laser-Doppler devices in the future to do more than display blood flow pictorially to aid decisions. In this paper, a probabilistic model is developed that would enable the predicted probabilities of time to heal within 14 days, 14–21 days or over 21 days to be displayed. The clinician would then have a prediction of healing time together with an indication of its probability of being correct. Eng-Kean Yeong *et al *(2005) [10] have predicted burn healing times using artificial neural networks and a reflectance spectrometer. The work here is in that spirit, but using a 'traditional' probabilistic model, the proportional odds (PO) model.

To enter a caveat: it is not yet practicable to use a mean flux-based methodology for clinical wound class prediction. This is because real burns have a distribution of flux values and the spatial distribution of these is also important. For these reasons two extra, overlap colors are used for the LDI image color-coding, instead of discrete class boundaries, to aid clinical interpretation with flexibility. At present, it is necessary to eyeball the flux levels and their spatial distributions, in conjunction with recognising confounding factors such as undebrided dead tissue (the dead epidermis not removed), to make an accurate assessment of wound healing potential.

The next section briefly describes the medical methodology, then the dataset used is described, then the statistical methodology is described and the results of fitting models to the data are given. Next, a statistical study of the 5-color palette currently in use is given, followed by conclusions.

## Methods

### Medical Methodology

The full methodology will be described in a separate clinical publication; briefly, the laser-Doppler imager records flux measurements of skin blood flow, measured in PU, for each of typically thousands of pixels corresponding to a wound area. The mean flux measurement with which we shall be largely concerned is the average flux across these pixels.

Burn wounds were assessed only once by laser-Doppler imager (moorLDI, Moor Instruments, UK) between 2 and 5 days post burn, and the burns were photographed at this time and also at 14 and 21 days post burn to record the burn at time of LDI and its subsequent healing.

Assessments were made at 14 and 21 days post burn because these times are important for clinical decisions on the burn treatment that is likely to result in least scarring: surgical (skin graft) or conservative (dressings only). A burn wound to most adults that heals within 21 days will do so with minimal scarring: 'Second degree burns that heal within 3 weeks are unlikely to leave scars' [11] ch5, p 70. Exceptions to this include infants, where the risk of infection is higher, and patients of ethnic origins predisposed to hypertrophic scarring. For these groups, surgical management is frequently performed on wounds that are expected to take longer than 14 days to heal [8].

Healing was assessed by clinicians and was defined as a wound with a continuous covering of epithelial cells. The boundary of healed wound at 14 days and 21 days post burn was assessed from the photographs taken at these times. Areas healed and not healed at 14 and 21 days were mapped back on to the original laser Doppler images for later analysis. This was necessary because a wound is usually heterogeneous, with parts healing at different times. These areas were then redrawn on a grey-scale photo image that was pixel-position identical with the flux image (obtained simultaneously with the flux image); flux values within the corresponding regions of interest were then exported for analysis.

Exclusions were made where burn wound boundary selections would have had a significant effect on the result, for example a boundary at a steep flux gradient. Prior to analysis, the data used here were screened for clinical factors such as wound infection, tattoos, drugs and concomitant patient sickness; and technical factors such as patient movement, edge effects and reflections.

The day of the LDI scan was determined by clinical factors, staff availability and the study protocol: the protocol restricted the day of scan to within 2 to 5 days post burn based on previous observations by others on the reliability of the LD technique.

Besides time to healing, the usual demographic variables, age and gender, were recorded, also skin pigmentation, which could affect recovery time. The medical history variables relevant to burns studies were total burned surface area, burn site on the body, and burn cause. Treatment and procedural variables were burn center and day of LDI scan.

The clinical decision to perform surgery inevitably censored the healing time in some cases. Some patients went to surgery for some of their burns before day 14 or between days 14 and 21. If the surgery was before day 14, the wound was excluded; if after day 14 we have recorded healing time > 14 days (similar to a patient who did not attend at day 21) unless there was further biopsy evidence. Where possible, biopsies were taken from wounds at surgery and some of these results for healing time have been included in the current analysis. Where the histological analysis found a full thickness wound, these are known to take longer than 21 days to heal; where histology found the wound to be superficial dermal (at the wound edge), these were classified as healing time < 14. Wounds found to be deep dermal were not included because these could heal before or after 21 days and even before 14 days if adjacent tissue had very high flux.

### Exploratory Data Analysis

In total, data on 768 burn areas were available, but of these only 310 had mean flux measured, of which 299 were complete, the other 11 having a missing outcome assessment because the patient did not attend one follow-up visit or because of surgery. The analysis here is focused on the 310 burn areas for which flux measurements were available. These areas came from 100 patients.

Table 1 summarises the univariate statistics, for the 310 cases for which flux data were available. Note that for one of the five burn centers, there were no cases with flux measured. Median age was 32 years, minimum 1 and maximum 88 years, and the %TBSA (total burned surface area) had median 6%, minimum 1% and maximum 68%. A larger sample of 581 burn areas, where flux measurements were not always available, was used to boost the statistics for an exploratory analysis of the relationships among the observational variables.

**Table 1:** Data summary, showing breakdowns of burn areas from 100 patients.

Variable	Category	Count
Healing	< 14 days	190

	14–21 days	47

	> 21 days	62

	censored	11

Burn center	1	4

	2	218

	3	37

	4	51

Scan day	2	60

	3	206

	4	37

	5	7

Burn site	limb	127

	extremity	88

	torso	72

	face	23

Gender	male	182

	female	128

Race	white	294

	black	16

Burn cause	scald	167

	flame	108

	chemical	10

	flash	12

	electrical	2

	contact	11

There are naturally many correlations among these variables. Demographic mix and %TBSA varied across the burn centers. Similarly, mean age varies with burn site.

There are some interesting gender differences. The type of burn site varies a little by gender, and this is statistically significant as shown by chi-squared test (*χ*^2^[3] = 11.7, *p *= 0.008), with men relatively more likely to be burnt on the face, and women on the torso. Burn cause varies considerably between the genders (table 2). It is mainly men who experience flash, chemical, electrical and contact burns, presumably because of gendered employment. This difference is very significant (*χ*^2^[5] = 75.6, *p *< 0.001.)

**Table 2:** Gender differences in burn statistics.

	Male	Female
Mean Age	33.4	32.0

Mean %TBSA	9.2	8.5

Burn cause		

Scald	49.7%	50.3%

Flame	61.1%	38.9 %

Chemical	100.0%	0.0%

Flash	100.0%	0.0%

Electrical	100.0%	0.0%

Contact	81.8%	18.2%

		

Healing time		

< 14 days healing	69%	31%

14–21 days healing	47%	53%

> 21 days healing	39%	61%

The unit of observation is individuals for age and %TBSA, otherwise it is burn area. 36% of the burn patients were female.

The age and %TBSA distributions are similar. It is also clear that healing time is longer for females than for males. There is however the problem of confounding, for example the slower female healing time could be at least partly the result of gender-specific burn sites and burn causes. This issue will be addressed in the survival-time modeling.

The slower female healing time is an important fact, and we briefly summarise previous findings in this area. There are a few studies on gender and morbidity, with mortality being the more frequent focus. The effect of gender, in these studies, is considered with other patient and wound variables: e.g. age, race, %TBSA and wound type, inhalation injuries and biochemical markers. Large %TBSA burns are included in the patient groups and these studies indicate that gender does influence outcome. Length of hospital stay and duration in intensive care have been used to assess morbidity. For adults, length of stay was greater for women [12] but in children, duration in the intensive care unit was found to be greater for boys [13]. The child gender difference was also found for mortality, higher for boys than for girls [14]. Adult mortality has been found to be greater in women by two-fold [15,16], and there is debate over the age group at highest risk [17,18].

The findings of the current investigation of the effect of gender on healing time are therefore consistent with previous work. However, it must be stressed that there are no studies looking at the residual effect of gender once the laser-Doppler measurement is known, which is the main focus of this study

### Statistical Methodology

There were 433 burn areas for which clinician's predictions of healing time using the LDI were available. Of these a subset of 310 wounds was appropriate for computing average laser-Doppler flux. The analysis here focuses on the use of flux as a predictor rather than using the clinician predictions, mainly because this shows the performance of the technology when quite divorced from clinical judgement. Also, the mean flux measurement data are probably better suited to answering questions about the role of covariates. Proportional-odds (PO) ordinal logistic regression is the most popular method of analyzing data such as these, where a dependent variable *Y *that takes ordered integer values is modeled as a function of a vector ***x ***of covariates. An introductory account is given in [19] and general descriptions in [20-22]. We seek to predict a dependent variable (healing time) that is ordered, and model the *logits *of the cumulative probabilities of healing in under 14 days or in 14–21 days as a linear function of the covariates. In the 'parallel lines' model usually fitted to the *k*th (of 2) cumulative probabilities *P*_1 _and *P*_2_, the logit is a function

(1) logit(*P*_*k*_) = log(*P*_*k*_/(1 - *P*_*k*_)) = *α*_*k *_+ ***β***^*T *^***x***,

where only the intercept *a *is a function of *k*, and the vector of coefficients ***β ***of the covariates ***x ***is not. The probabilities of the three healing times, *p*_1_, *p*_2 _and *p*_3 _are given by *p*_1 _= *P*_1_, *p*_2 _= *P*_2 _- *P*_1 _and *p*_3 _= 1 - *p*_1 _- *p*_2 _= 1 - *P*_2_.

Surgeons occasionally desire to predict probabilities of healing over different time intervals than the ones used here. We suggest that this could be attempted by regarding *α *as a function of time *t*, for example *α *= *γ*log(*t*/*t*_0_). Then we can determine *γ *and *t*_0 _from *α*_1 _= *γ*log(14/*t*_0_), *α*_2 _= *γ*log(21/*t*_0_). With this parameterization, probabilities of healing over two or three different intervals could be found. This model corresponds to a log-logistic distribution of healing time for fixed covariates.

In a later section of this paper, on palette derivation (a discriminant problem) we follow the methodology of [23,24], which dispenses entirely with the need to choose an ordinal model. However, for assessing the significance of covariates using likelihood-based inference, a model is required. Other models besides PO are also available ([25-27]), and there is no overriding reason to choose the PO model. Hence two other models were also fitted, to see whether the PO model could be improved on, the probit and continuation ratio (CR) models [26,28]. The latter model is applicable where, as here, the three data groups are periods. As will be seen, neither of these models fitted better than the PO model. Other models, such as the multinomial model, are useful only when the categories are unordered, and are less efficient than ordinal models when used with ordinal data [29].

The covariates used in this study, besides the LD flux measurement, are those demographic variables that are usually important in medicine, age and gender, plus those known to be important in predicting healing time, such as %TBSA and burn site, and some that might possibly be thought to be relevant, such as skin pigmentation and burn cause. It is thought that these last two are probably not important. For example, natural skin pigmentation does not affect LDI flux from debrided wounds because the pigment is removed with the epidermis.

The proper selection of predictor variables for use in a model raises some statistical problems (e.g., [28], ch. 4. In particular, modeling choices made by the statistician after viewing the data are often not reflected in the final p-values and confidence intervals produced. Results then appear unduly significant or accurate, and traditional approaches, such as stepwise regression, can give misleading results. This problem looms large when sample sizes are small and there are many variables. Here, fortunately, we have the opposite situation.

The data posed some problems that necessitated a purpose-written computer program. An example is the existence of a few censored cases, where patients did not attend a hospital visit. In these cases, it was known only that healing took place in under 21 days or after more than 14 days. Where surgery was performed, burn severity information was obtained from biopsy results: less than 14 days was assumed for superficial dermal wounds; more than 21 days was assumed for full thickness wounds. Fitting the model by maximum-likelihood estimation meant that these cases could be included. A more serious problem was the existence of multiple burn areas on the same individual. The 310 burn areas occurred on only 100 patients. Although demographic variables that could influence healing time such as age and gender were known, there might be a further 'frailty' that varied between individuals, so that healing times would not even be conditionally independent [30]. Obesity and smoking, for example, affect health, but were not tested here. To model frailty, we add a hypothetical frailty variable, and each individual has a random value of it. A normal distribution for the variable is the simplest choice, and as frailty turned out to be a small effect, no more elaborate modeling was done.

Taking the mean contribution from this variable as zero and the variance as *σ*^2 ^gave a likelihood function

(2) $$ℒ=\prod_{i=1}^{N}\frac{1}{\sqrt{\left(2\pi \sigma^{2}\right)}}\int_{-\infty }^{\infty }\{\prod_{j=1}^{n_{i}}\text{Prob}(Y_{j}|\alpha_{1},\alpha_{2},\beta^{T}x_{j}+z)\exp(-z^{2}/2\sigma^{2})\}\text{d}z,$$

where the *j*th burn area on the *i*th of *N *individuals has healing time *Y*_*j *_and covariate vector **x**_*j*_. The assumption of a zero mean is nugatory, as a nonzero mean would be absorbed into the constants *α*_1 _and *α*_2_. For censored data, where for example, healing is only known to occur in under 21 days, the probability in (2) is the probability of this observed event, *p*_1 _+ *p*_2 _or *P*_2_.

The integration could be done by the Gauss-Hermite method as recommended by [31]; in fact adaptive integration using the Numerical Algorithms Group (NAG) integration subroutine D01AMF was used here. The website [32] gives full details of the NAG routines mentioned here.

The log-likelihood function ℓ = ln ℒ was maximised using the NAG function minimisers E04UCF and E04CCF [32]. The former uses a sequential quadratic programming method and is relatively fast, and the latter uses the Nelder-Meade method and is slow but robust. The best of a large number of random restarts (20) from the current function maximum was used, to ensure that the global maximum of the log-likelihood function had been found.

The asymptotic covariance matrix for fitted model parameters is taken as the inverse of the Hessian matrix $-\partial^{2}\ell /\partial \beta_{i}\partial \beta_{j}{|}_{\beta =\hat{\beta }}$ where the *β*_*i *_are model parameters, and $\hat{\beta }$ are the maximum-likelihood estimates. To obtain a more accurate estimate of parameter errors for small samples, the likelihood was maximised for 1000 bootstrapped samples, in which individuals were sampled from the dataset with replacement; see e.g. [33]. Bootstrap resamples that did not allow all parameters to be estimated were rejected. This can happen, if for example, the resample does not contain any examples of a particular burn cause.

Estimates of the model's predictive ability were found by taking the predicted healing time as that with the highest probability. The resulting percentage of correct assignments is however liable to be over-optimistic, because the model is evaluated in-sample. A recommended method of correcting for this is to calculate the 'optimism' of an in-sample estimate as the mean of a large number of differences *D*_*i *_between estimates from a bootstrapped dataset evaluated on the bootstrapped dataset and on the original dataset. Finally, the mean optimism $\bar{D}$ is subtracted from the sample estimate (e.g. [28], section 5.2.5, [33]). This procedure is better than simply splitting the sample into two, one for model fitting and one for validation [34]. The predictive ability of a linear model may be measured by the coefficient of determination, *R*^2^. For instance, [35] and others proposed a pseudo *R*^2 ^for general models given by

$$-\log(1-R^{2})=\frac{2}{n}\{\ell (\hat{\beta })-\ell (0)\},$$

where ***β ***= **0 **denotes the 'null' model. Nagelkerke (1991) [36] proposed a correction, since the maximum value of *R*^2 ^attainable is less than 1. The correction required normalising *R*^2 ^to its maximum value of 1 - exp{2*n*^-1^ℓ(**0**)}, and we use this corrected value of *R*^2^.

Significance tests that a parameter *β*_*i *_is zero would classically be done using the Wald statistic, i.e. using the estimated standard error of the parameter estimate (see e.g. [28], section 9.2.2). Alternatively, the increase in log-likelihood Δℓ on 'floating' the parameter can be used as a test statistic, when under *H*_0 _we have that 2Δℓ ~ *χ*^2^[1]. An alternative is to carry out a Wald test using the bootstrapped error estimate. However, an exact test can be obtained by retaining Δℓ as a test statistic, but obtaining its reference distribution under *H*_0 _by permuting the relevant variable *x*_*i *_among the cases. The p-value of the test is the proportion of permutations for which the computed Δℓ exceeds the value for the original sample (see e.g. [37] and [33], chapter 15.). Variables such as gender must of course be permuted among individuals rather than among burn areas. The logic is that under *H*_0 _gender is irrelevant, and so the permuted datasets generate the reference distribution for the test statistic. Note that this is not the more elaborate procedure used by [38].

Since the number of distinguishable permutations (combinations) is very large, a random sample of 1000 was generated to compute the p-value. A random permutation of *n *labels held in an array was achieved by swapping each array element in turn with a random array element.

The main advantage of a permutation test over a bootstrap hypothesis test is that the permutation test correctly generates the reference distribution of the test statistic under *H*_0_, whereas the bootstrap hypothesis test can only generate the distribution of the test statistic under *H*_1_. Also, the p-value obtained is exact, and not an asymptotic approximation. Permutation tests have a simple rationale; under *H*_0_, gender labels are irrelevant to healing, and so we can obtain an equivalent dataset by shuffling them. The only drawback here is the extra computing time needed; a separate set of permutations must be carried out for each variable or related set of variables in turn.

In the analyses done here, variable selection was not a problem: given the laser-Doppler flux, only gender was significant. This contrasts with many clinical studies, where many covariates are significant, and the choice of best model is not easy.

## Results

### Data Analysis: LDI performance

#### Predictors of healing time without LDI measurements

Before considering the laser-Doppler measurements as predictors of healing time, it is worthwhile modeling healing time purely as a function of demographic and observational variables. This allows the partial confounding caused by correlation between covariates to be resolved, and in particular answers the question of whether the slower female healing time is solely due to gendered burn site type. Table 3 shows the results of a proportional odds model analysis, using the model from (1) based on 581 regions of interest from 130 individuals. The variables ***x ***were dummy variables corresponding to 3 of the 4 burn sites, gender, race, burn cause, and two variables for age effect. The reference categories were burn site 1 (limbs) and scald for burn cause. Analyses were also done including factors such as burn center and day of scan, but any effects due to these were not significant, and table 3 shows an analysis including only the more interesting variables. Note that the joint significance of related variables such as linear and quadratic age terms was also evaluated by likelihood ratio tests (not shown).

**Table 3:** Demographic and observational predictors of healing based on 581 burn areas, where se* is the bootstrapped standard error of the estimated parameter.

Parameter	$\hat{\beta }$	se	se*	p-value 1	p-value 2
*α*_1_	1.209	0.267	0.532	.041	0.023

*α*_2 _- *α*_1_	1.008	0.095	0.122	< .001	< .001

Burn site 2 (extremity)	0.912	0.225	0.361	.018	0.012

Burn site 3 (torso)	0.370	0.250	0.300	.21	0.217

Burn site 4 (face)	1.732	0.469	1.18	.023	0.141

Gender (female)	-0.696	0.197	0.389	.084	0.073

Race (black)	0.168	0.587	2.34	.888	0.943

Age (yrs)	-0.0084	0.0041	0.0072	.236	0.239

(Age - 35)^2^	-0.0006	0.0002	0.00034	.106	0.103

%TBSA	-0.0104	0.0088	0.043	.736	0.762

Burn cause: flame	-0.589	0.219	0.370	.098	0.111

Burn cause: chemical	-0.847	0.503	1.97	.351	0.668

Burn cause: flash	-0.385	0.436	0.772	.617	0.618

Burn cause: electrical	-2.175	0.964	8.49	.687	0.798

Burn cause: contact	-1.507	0.511	1.36	.143	0.266

The two bootstrap p-values from the two-sided bootstrap hypothesis test are from the bootstrap percentile and bootstrap parametric tests described in the text. The more reliable p-values derived from permutation tests are given in the text.

It can be seen that the bootstrapped standard errors of parameter estimates are always larger than the asymptotic theory values that are commonly used. This is particularly marked for variables such as race and some burn causes, where very few individuals may have a particular burn cause. This shows the wisdom of using bootstrapped estimates rather than relying on asymptotic theory. The p-values in the tables are those of two simple bootstrap hypothesis tests. The first is the bootstrap percentile test, where the resampled distribution of fitted parameters is shifted left by $\hat{\beta }$ so that it approximates to the distribution of the fitted parameter values under *H*_0_. This gives a simple (nonparametric) two-sided bootstrap hypothesis test. The second p-value is derived using the normal approximation and the bootstrapped standard error se*. The analytic approximation sometimes gave much lower p-values than either of these tests; for example, for gender, the analytic p-value was 0.0004, as compared to the last two columns of table 3 (p-values of 0.084 and 0.073).

The Cox and Snell *R*^2 ^= 0.138, the Nagelkerke *R*^2 ^= 0.158, and the proportion of correct assignments is only 61%, showing the poor predictive ability of this model.

**Figure 3:**
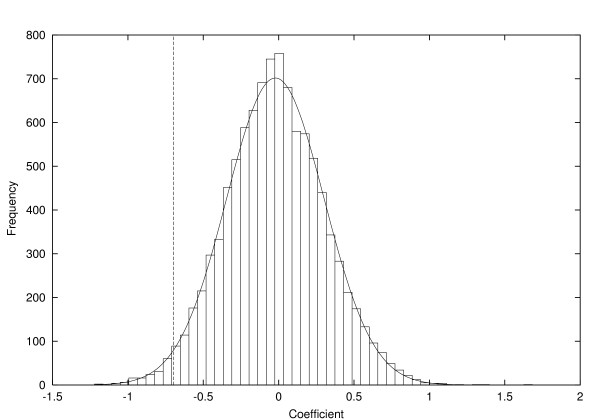
**The coefficient $\hat{\beta }$ (vertical dotted line) and its permutation distribution under *H*_0 _for gender, in the absence of a laser-Doppler measurement, with fitted normal distribution**.

Healing appears much faster at some burn sites such as extremities and face, and permutation tests showed that the variation in healing time between burn site types is very significant (*p *< 0.001). Gender was also significant (*p *= .026). Figure 3 shows the coefficient $\hat{\beta }$ for gender and its reference distribution derived by permutation of gender labels, together with a fitted normal curve. Age was also significant (*p *= .024). The effect of age is in the table modeled using a linear and quadratic term, the quadratic term being (age - 35)^2 ^rather than age^2 ^for numerical stability. These two terms give a healing time that improves slightly with age up to about age 30, and subsequently decreases. Modeling the effect of age using dummy variables corresponding to age ranges gave similar results. Burn cause was not significant (*p *= 0.194). Burn center and day of scan were also not significant factors.

#### Predictions of healing time with LDI measurements

Table 4 presents the results when the laser-Doppler flux measurement is included. Permutation tests show that burn site is now not significant (*p *= 0.78), and neither is age (*p *= .24). However, gender is still a significant predictor (*p *= 0.002), and its statistical significance has increased. Aside from gender, the laser-Doppler measurement is a sufficient statistic. The Cox and Snell *R*^2 ^= 0.931 and the Nagelkerke *R*^2 ^= 0.976, confirming the excellent predictive ability of this model.

**Table 4:** Predictors of healing, when LDI mean flux measurement is included, based on 310 burn areas for which flux measurements were available.

Parameter	$\hat{\beta }$	se	se*	p-value 1	p-value 2
*α*_1_	-0.292	0.764	1.115	0.77	0.79

*α*_2 _- *α*_1_	5.826	0.912	1.515	0.008	0.00012

Burn site 2 (extremity)	0.635	0.626	0.685	0.34	0.35

Burn site 3 (torso)	0.192	0.705	0.956	0.79	0.84

Burn site 4 (face)	0.580	1.384	8.19	0.78	0.94

Gender (female)	-1.979	0.597	0.793	0.023	0.012

Race (black)	-1.579	1.373	5.99	0.376	0.792

Age (yrs)	-0.0002	0.012	0.014	0.993	0.990

(Age - 35)^2^	-0.0008	0.0004	0.00094	0.191	0.280

%TBSA	0.048	0.039	0.048	0.272	0.3248

Log of mean flux	9.367	1.190	2.35	.012	0.00007

Meaning of headings as for table 3.

Including a random effect as in (2), when gender is omitted and only mean flux is used as a predictor, a drop in minus log-likelihood from 73.56 to 69.21 is obtained on including the random effect, which is thus clearly significant (p-value of significance test is 0.0016). The standard error of the random effect was estimated as 1.91. However, on including gender as a covariate, minus the log-likelihood then fell from 63.44 to 62.10, giving a non-significant random effect (p-value of 0.052). The standard error was 1.38, a little lower. It seems that once gender is included as a covariate, the random effect is no longer needed. This suggests that covariates that retard healing and were not available in the dataset, such as smoking and obesity, might also be irrelevant once blood flow measured as mean flux is known.

To test whether the gender effect varied with age, further terms were added to the model, such as an age by gender interaction term (equal to age for females, zero for males). A permutation test was carried out, by permuting this interaction variable among individuals and finding the proportion of such random permutations for which the log-likelihood was at least as large as the observed value, when this variable was included in the model. The p-value was 0.105, showing that there is no evidence that the gender difference in healing times varies with age. Finally, we considered use of maximum and minimum logged flux, standard deviation of flux (coefficient of variation), and higher powers of the logged flux. None of these variables were statistically significant.

**Figure 4:**
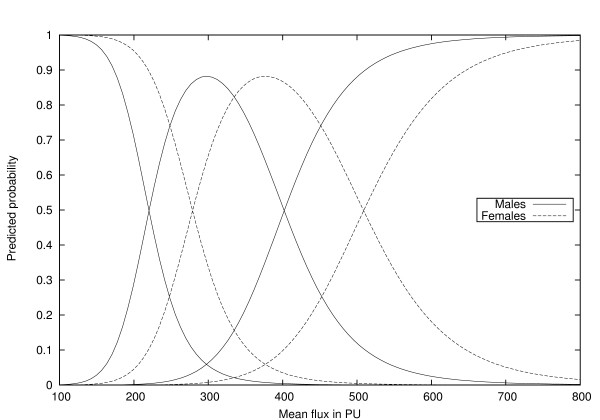
**Model predictions for probabilities of slow, medium and fast healing for males and females under the PO model, as a function of mean laser-Doppler flux**.

The model finally arrived at is very simple. Including gender besides mean flux as a predictor, table 5 shows the crosstabulation of predicted and observed healing times. As can be seen, the bootstrap-corrected percentage of correct classifications is 92%. This compares to the situation where gender is omitted from the model, when the correct classification rate is 91.3%, reducing to 90.9% when the bootstrap 'optimism' correction is applied. Figure 4 shows the predicted probabilities of the three healing periods as a function of mean flux.

**Table 5:** Classification performance of the PO model with gender and mean flux.

	Pred. < 14 days	Pred. 14–21 days	Pred. > 21 days
Obs. < 14 days	182	8	0

Obs. 14–21 days	9	36	2

Obs > 21 days	0	3	59

The % correctly classified is 92.6, the bootstrap 'optimism' is 0.6% and the corrected % correctly classified 92.0%.

Varying the model used from the proportional-odds model had very little effect. Using the probit link function instead of the logit decreased the log-likelihood function by 1.16, a slightly worse fit, and changing to the continuation ratio model decreased the log-likelihood by 0.207. The justification for taking the PO model with gender and mean flux as covariates as the definitive model was that it was the minimum-AIC model (MAICE model).

#### The effect of total burned surface area (%TBSA)

Since the effects of total burned body area and skin pigmentation are likely to be important issues in the use of LDI, the statistical conclusions here will be spelt out. In the case of skin pigmentation, there are only 15 burn areas from black-skinned patients. It is not surprising that the coefficient for this factor is not statistically significant, and we really cannot draw any conclusion here from the data. There is however no reason to think that skin pigmentation would have an effect on healing for a given observed flux once skin is debrided.

The case of percentage total body surface area burned is slightly different. Here, apart from a few cases, the data do not extend much beyond 30% TBSA. In fact, the distribution of %TBSA fits well to a lognormal distribution, so that the natural logarithm of %TBSA has mean 1.956 and standard error 0.883.

However, this is a wide variation, and it allows the %TBSA coefficient in equation 1 to be determined with some accuracy; from table 4, the estimated coefficient is 0.0481, with a standard error of 0.051. Note that the sign of the effect is such that survival would improve with %TBSA, which would seem unlikely, so there is not a shred of evidence for any %TBSA effect.

A statistical task remains however, because the relevant question is slightly different. Given the possible (posterior) distribution of the %TBSA effect as determined from the data, would any clinically significant degradation in predictive performance result at high %TBSA from assuming a model with zero %TBSA effect?

To give some answer to this, a fortran95 simulation program was written. Fluxes were simulated by 'bootstrapping' the flux measurements (sampling with replacement), and a sample of 'patients' were generated with PU (perfusion unit) measurements from this distribution, and random %TBSA from the lognormal distribution of %TBSA. Healing times were simulated from the average flux model prediction for healing time, using flux and %TBSA as predictors. The 'data' healing times were generated randomly from the model with the coefficient of the %TBSA effect randomly chosen from its normal distribution. This can be done, as we know the estimated coefficient and its standard error. We thus imagine that the data will obey an unknown model that has a %TBSA coefficient consistent with our findings. The model used for prediction however has the %TBSA coefficient set to zero.

The misclassification rate obtained when the maximum likelihood estimate of healing time was used (i.e. we took the largest of the 3 probabilities) was 10.2% in this simulation. This agrees well with the 9% misclassification rate seen in the simple fit of the model to data. On moving to a scenario where the lognormal distribution of %TBSA is scaled up so that the mean %TBSA is 40% the misclassification rate increased to 13.6% and with 75% mean %TBSA, the misclassification rate increased to 17.7%.

This shows that, as expected, uncertainty in the amount of dependence of healing time (given a flux measurement) on %TBSA, does introduce extra error into healing time predictions as we extrapolate to high average %TBSA. Although we cannot reject the hypothesis that %TBSA has no effect on healing time once given a flux measurement, there could still be an effect large enough to degrade prediction ability when predictions are made ignoring this covariate. The misclassification rate could increase by a factor of 1.7. However, for healing time, this would only give a drop to maybe 85% accuracy. The conclusion is that, unless qualitatively different phenomena occur for patients with high %TBSA, the degree of accuracy of the model parameter estimates rules out any large increase in the rate of misclassification.

#### Goodness of Fit

This was explored in two ways: by fitting various models in the attempt to improve fit, and by diagnostic plots. The first approach led to the use of the logged mean flux as the best predictor in proportional-odds models.

**Figure 5:**
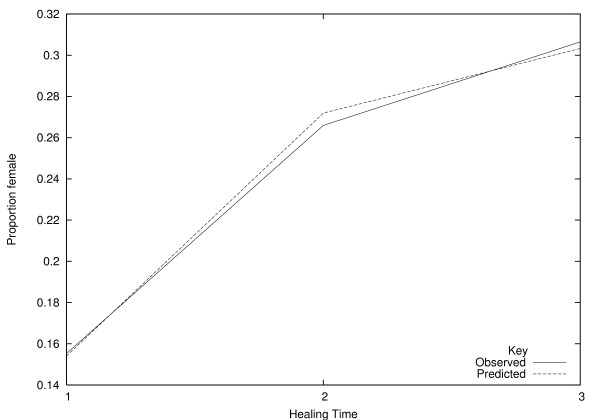
**Observed and predicted proportions of females, under the proportional odds model**.

**Figure 6:**
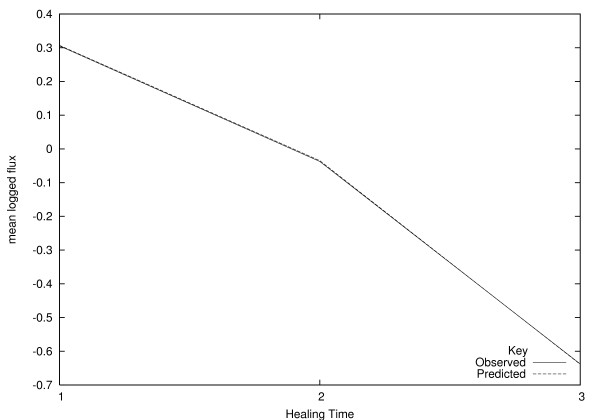
**Observed and predicted mean logged flux (PUs), under the proportional odds model**.

Figures 5 and 6 show observed mean values for predictor variables for the three healing classes, and the predicted mean values from the PO model. Predicted mean values for a variable are calculated by including all cases, weighted by the predicted probability of group membership (e.g. [28], section 13.3.5). This relies on the use of Bayes' theorem, to turn the model into a predictive model for the covariates, given the healing time group. A bad fit is indicated by a strong divergence between the observed and predicted values, or an observed value that does not change monotonically with healing time. In figures 5 and 6, the agreement looks very good. The high value of *R*^2 ^and the high predictive ability confirm that the model fits the data well.

### Palette Construction

Recall that a false color image of the burn area is used by clinicians, as shown in figure 1. The 'palette' is the choice of PU-cutpoints that defines whether the pixels of a burn region should be colored with color 1, 2, 3, 4 or 5, and does not specify which shades are actually used.

The 5-color palette currently in use has PU-cutpoints at 200, 260, 440 and 600 PU. The colors 1 to 5 are blue, green, yellow, pink and red, and in fact the blue region is divided into dark and light blue at 140 PU. The decision rule for healing-time prediction recommended to clinicians is that the 'buffer colors' green and pink should be lumped in with whichever of their neighbors has the larger area (rule 1).

Pixel data were available for two datasets, one used in palette construction, the other for palette validation. These were combined for the analysis here. The statistical analysis aimed to appraise the palette in current use, and to discover whether it could be optimised. Also, with an eye to the future, with individual pixel data available, we ask what is the best decision rule that can be constructed for 'automatic' use. Here, the device envisaged would show the probabilities of the three healing times for a region of interest. It is interesting to ask whether the mean flux that was used earlier as a healing-time predictor is a sufficient statistic, or whether, given the full distribution of flux density, a better predictive rule could be found. Using two flux cutpoints, the optimum was found by minimizing the mean percentage of wrong healing-time class assignments per healing-time group. The NAG function Nelder-Meade function minimizer E04CCF was used [32], with 50 sets of random starting values. It is also possible to convert this to a discrete optimization problem, by varying the two cutpoints in units of 1 PU. This gave the same results.

#### Appraising the palette

Table 6 shows how the current palette would perform, if the cutpoints were chosen in the center of each of the two 'buffer' colors. The decision rule based on the proportions of pixels below cut 1 (*q*_1_), between the cuts (*q*_2_) and above cut 2 (*q*_3_) is

**Table 6:** Classification performance of: (A), the current palette, taking color boundaries in the center of 'buffer' colors at 230 and 520 PU.

A) **Current**	Pred. < 14 days	Pred. 14–21 days	Pred. > 21 days
Obs. < 14 days	160	26	0

Obs. 14–21 days	5	39	3

Obs > 21 days	0	3	25

	% wrong	% av. wrong per class	

	14.2	13.9	

			

B) **Optimized**	Pred. < 14 days	Pred. 14–21 days	Pred. > 21 days

Obs. < 14 days	175	11	0

Obs. 14–21 days	8	36	3

Obs > 21 days	0	3	25

	% wrong	% av. wrong per class	

	9.6	13.3	

			

C) **Mean flux**	Pred. < 14 days	Pred. 14–21 days	Pred. > 21 days

Obs. < 14 days	176	10	0

Obs. 14–21 days	7	38	2

Obs > 21 days	0	3	25

	% wrong	% av. wrong per class	

	8.4	11.8	

Note that the current palette was not designed to be used in this way! For (B), the optimized palette, the flux boundaries are 241 and 452 PU. Lastly, (C), using mean flux as a classifier, flux boundaries are 250 and 465 PU.

if(*q*_1 _≥ *q*_2 _&*q*_1 _≥ *q*_3_)group = 3

if(*q*_2 _≥ *q*_1 _&*q*_2 _≥ *q*_3_)group = 2

if(*q*_3 _≥ *q*_1 _&*q*_3 _≥ *q*_2_)group = 1.

When the five colors are used as designed, the classification of a case depends on the rule used for interpreting buffer colors. We can formulate at least four possible decision rules. Rule 1, 'lump buffer colors in with their neighboring color of larger area' is what is recommended to clinicians. Rule 2 is to ignore the buffer colors, and to predict healing-time from the largest area of the three colors blue, yellow and red. Rule 3 is to use the buffer colors to make the best case for each healing time. The visual decision made by a clinician would then be for example 'is the total area of color consistent with slow healing (colors 1 and 2) greater than the total area consistent with medium-time healing (colors 2, 3 and 4)?'. This is illustrated in the pseudocode:

if(*q*_1 _≥ *q*_3 _+ *q*_4 _&*q*_1 _+ *q*_2 _≥ *q*_4 _+ *q*_5_)group = 3

if(*q*_2 _+ *q*_3 _≥ *q*_5 _&*q*_3 _+ *q*_4 _≥ *q*_1_)group = 2

if(*q*_5 _≥ *q*_2 _+ *q*_3 _&*q*_4 _+ *q*_5 _≥ *q*_1 _+ *q*_2_)group = 1.

Rule 4 is 'regard the buffer colors as identical to the neighboring color corresponding to faster healing time'. Dark and light blue then mean slow healing, green or yellow mean medium healing time, and pink or red mean fast healing. This means that the 6-color palette is effiectively used as a 3-color palette.

Table 7 shows the performance of the palette under these four rules. It is interesting to see that the accuracy of diagnosis is very similar under rules 1, 2, and 3. This is reassuring, as no doubt, regardless of advice, different clinicians will interpret the false-color image in their individual ways. However, adoption of rule 4 would give worse results. From table 6 the optimized palette with 3 colors gives only a tiny improvement in the average percentage of wrongly-categorized cases. The cutoff between fast (< 14 days) healing and medium (14–21 days) healing has moved down slightly, so that fewer fast-healing cases are misdiagnosed as medium-healing time cases, but on the other hand, more medium healing-time cases are misdiagnosed as fast healing. In general, it was found by varying the cutoffs that many 5-color palettes could be devised with similar performance. The buffer zones can be wider or narrower. The optimized palette in table 7 performs only slightly better than the palette in current use, and some of this improvement may be due to the double use of the data for fitting and validation. In fact, using the leave-one-out method of crossvalidation, the 11.6% misclassification rate increased to 13.7%, eroding the improvement completely.

**Table 7:** Classification performance of the current palette (A) under allocation rules 1–2, (B), under rule 3, and (C) under rule 4, and (D) of the optimized palette under rule 1.

A) **Rules 1 and 2**	Pred. < 14 days	Pred. 14–21 days	Pred. > 21 days
Obs. < 14 days	162	24	0

Obs. 14–21 days	5	39	3

Obs > 21 days	0	3	25

	% wrong	% av. wrong per class	

	13.4	13.6	

			

B) **Rule 3**	Pred. < 14 days	Pred. 14–21 days	Pred. > 21 days

Obs. < 14 days	161	25	0

Obs. 14–21 days	5	39	3

Obs > 21 days	0	3	25

	% wrong	% av. wrong per class	

	13.8	13.7	

			

C) **Rule 4**	Pred. < 14 days	Pred. 14–21 days	Pred. > 21 days

Obs. < 14 days	177	9	0

Obs. 14–21 days	11	35	1

Obs > 21 days	0	7	21

	% wrong	% av. wrong per class	

	10.7	18.5	

			

D) **Optimized**	Pred. < 14 days	Pred. 14–21 days	Pred. > 21 days

Obs. < 14 days	178	8	0

Obs. 14–21 days	7	36	4

Obs > 21 days	0	2	26

	% wrong	% av. wrong per class	

	8.1	11.6	

This has flux boundaries 188,311, 353 and 601 PU.

Clearly, other criteria than the percentage of wrong diagnoses would be needed to optimize the 5-color palette. A possible approach would be to penalize certainty in wrong diagnosis, and uncertainty in right diagnosis. It is arguably best to be right, and to be sure that you are right, less good to be right but unsure, worse to be wrong and unsure, and worst of all to be wrong and sure that you are right. The probability *q*_1 _+ *q*_3 _+ *q*_5 _is a measure of certainty, and *q*_2 _+ *q*_4 _a measure of uncertainty. A loss function such as

$$\sum_{\text{wrong class}\text{.}}(1+q_{1}+q_{3}+q_{5})+(1/10)\sum_{\text{right class}.}\left(q_{2}+q_{4}\right)$$

penalizes complete certainty in wrong diagnosis as twice as bad as a wrong diagnosis with complete uncertainty, and a right diagnosis with complete uncertainty is 1/10 as a bad as the best wrong diagnosis. Minimizing this loss function, the PU cutoffs were 191, 243, 326 and 699 PU. The penalizing of uncertainty in diagnosis would allow an 'automatic' method of palette construction. Unfortunately, this resembles the problem of utility maximisation in healthcare and elsewhere; we could devise optimum policies if we could specify our utility function. Here, one could perhaps survey burns surgeons to elicit their preferred loss function, and only then could a palette be computer-generated. The value of the work here is that it has demonstrated that a 5 or 6-color palette cannot simply be derived automatically, in the same way as can a 3-color palette, but that further judgements are required.

#### Automatic decisions

Disregarding the clinical complications, for example ensuring that a region of interest is sufficiently homogeneous in blood flow for the entire region to heal at the same rate, we seek the best automatic decision rule. Table 6 shows that a rule based on the mean flux slightly outperforms rule 1. This is an example of the ODAO (Optimal Discrimant Analysis for Ordinal responses) method described by [23] and [24]. They found that this method outperforms other discriminant methods, because there is no need to model the functional form of the relation between (here) mean flux and healing time; one simply chops the mean into three ranges. Using the median flux or the mean logged flux gave slightly worse performance, and several other methods that were tried did not perform as well. It does indeed seem that the mean flux in a homogeneous burn region is the best predictor of healing time, and that the shape of the flux distribution is irrelevant. Clinically, this can make sense because mean blood flow in an area is proportional to total blood flow.

## Discussion

The high accuracy of LDI, over 90%, relative to the 70% accuracy achievable by unaided clinical judgement, and the fact that the only demographic variable influencing healing time once the laser-Doppler measurement is known is gender, are the main results of the analysis; it is significant that age and %TBSA did not aid prediction of healing time. Also, it has been established that the mean flux over an area is a 'sufficient' flux statistic; it is impossible to obtain better predictive power by substituting the median flux, by adding standard deviation, or by using quantiles of the flux distribution.

The accuracy of LDI seen in this study is lower than the levels reported by clinicians ([5,6,8] etc) and could be because the current assessment is based on *only *LDI whereas the other studies would have included clinical judgement. Also, instead of the 3-way classification of healing time used here, frequently only a 2-part classification is use: treat surgically or not. Only 6.5% of cases are misclassified by the methodology used here under that scheme.

Women tend to heal more slowly than men even when the blood flow as measured by laser-Doppler flux is corrected for. It would therefore be possible to obtain a slight improvement (1.1%) in classification accuracy by using knowledge of gender in addition to the laser-Doppler measurements.

Turning to the %TBSA results, this is an unusual statistical situation, where despite a non-significant result, we seek to explore the possible degradation of predictive ability at high %TBSA resulting from our uncertainty over the effect of %TBSA. Clearly, we have been able to transform a statement of 'no evidence for an effect' into a statement about the possible degradation of performance arising from the uncertainty over the size of the effect. However, any really believable evidence that LDI can be used up to high values of %TBSA must come from further clinical trials.

With an eye to the future, use of a probabilistic model and its incorporation into the laser-Doppler imaging software would enable clinicians to see when the healing time prediction was very reliable and likely to be correct, and when the diagnosis was less certain. The best model obtained of the probabilities *p*_1_, *p*_2_, *p*_3 _of fast, medium, and slow healing times is, for completeness:

p1=11+exp⁡(−α1−βgG)/yβf,p2=11+exp⁡(−α2−βgG)/yβf−p1,p3=1−p1−p2,

where *y *= exp(-6)$\bar{x}$, $\bar{x}$ is mean flux in PU, *G *is gender (0 for male, 1 for female), *α*_1 _= .0222, *α*_2 _= 5.563, *β*_*f *_= 9.185, *β*_*g *_= -2.164. The factor of exp(-6) was added for numerical reasons.

Uncertainty over healing times is conveyed visually by using a 5-color palette instead of the three colors needed for decision making. The statistical investigation into the derivation of an optimum palette has shown that the widths of the two 'buffer color zones' are not fixed by the requirement that the percentage of wrong healing-time diagnoses be minimized. They could be fixed by penalizing 'certain' wrong diagnoses more than uncertain ones. To construct an 'optimum' palette, it would be necessary to survey burn surgeons to elicit the best form of loss function to be used. Further work may be carried out along these lines.

## Conclusion

This study has shown how contemporary statistical techniques can be used to assess performance of a medical imager and to reveal which clinical factors are important with and without use of the device. Ease of image interpretation is essential for such devices. In this respect, it has been shown that statistical techniques alone are incapable of providing a solution and that clinician's input is required.

## Competing interests

The authors declare that they have no competing interests.

## Authors' contributions

The first author carried out the statistical analysis, and the other authors set up the clinical investigation, obtained the clinical results and provided clinical information.

## Pre-publication history

The pre-publication history for this paper can be accessed here:

http://www.biomedcentral.com/1471-2288/9/11/prepub
